# Availability, effectiveness and safety of cadaveric and fresh allogeneic skin grafts in pediatric burn care—a review

**DOI:** 10.1007/s10561-025-10161-8

**Published:** 2025-03-15

**Authors:** Moritz Lenz, Nikki Allorto, Shobha Chamania, Clemens Schiestl, Christoph Mohr, Michael Boettcher, Julia Elrod

**Affiliations:** 1 Department of Trauma and Orthopaedic Surgery, University Medical Center Hamburg-Eppendorf, Hamburg, Germany; 2 Department of Pediatric Surgery, University Medical Center Hamburg-Eppendorf, Hamburg, Germany; 3 Pietermaritzburg Burn Service, University of KwaZulu-Natal, Pietermaritzburg, KwaZulu-Natal South Africa; 4 Department of Burn Surgery, Choithram Hospital and Research Centre, Indore, India; 5 Department of Surgery, Plastic and Reconstructive Surgery, Pediatric Burn Center, University Children’s Hospital Zurich, Zurich, Switzerland; 6 Department of Pediatric Surgery, University Medical Center Mannheim, Heidelberg University, Theodor-Kutzer-Ufer 1-3, 68167 Mannheim, Germany

**Keywords:** Burn, Scald, Cadaver skin, Allografts, Homologous skin transplantation, Pediatric, Children

## Abstract

Burn injuries in children are a critical public health issue with significant mortality and morbidity. Allogeneic skin grafts, both cadaveric and freshly donated, have been utilized in pediatric burn care since many years, yet their efficacy and safety remain to be systematically assessed. This systematic review (PROSPERO number: CRD42024560654) analyzed studies from 01/2000 to 07/2024 sourced from PubMed. Inclusion criteria targeted RCTs and retrospective studies focused on the use of allogeneic skin grafts in pediatric burn patients. Extracted data were presented in a narrative synthesis and a comprehensive table. Established tools were used for risk of bias assessment. 13 studies were deemed suitable for analysis, with only two qualifying as RCTs. Allogeneic skin grafts have shown promise in managing pediatric burns, especially in resource-limited settings where autografts or skin substitutes are not available. Studies varied in their treatment approaches, with allogeneic grafts often used for more severe burns, suggesting that observed adverse effects may be due to injury severity rather than treatment type. The retrospective nature of the majority suggests a limited level of evidence. Moreover, the heterogeneity among study designs and patient populations makes it difficult to draw definitive conclusions. Allogeneic skin grafts represent a valuable treatment option in pediatric burn care. However, further well-designed RCTs are essential to establish a stronger evidence base for their use and to guide clinical decision-making. The current literature underscores the potential of allogeneic grafts but also the necessity for more nuanced research tailored to pediatric needs.

**Supplementary Information:**

The online version contains supplementary material available at 10.1007/s10561-025-10161-8.

## Introduction

Thermal injuries rank among the most severe traumas and are often associated with high mortality and morbidity (Peck 2011). Children are particularly susceptible due to their explorative nature and limited risk awareness (Abarca et al. 2023; Armstrong et al. 2021; Johnson et al. 2017). Incidence rates are tenfold higher in low- and middle-income countries (LMIC) compared to high-income countries (HIC) (Peck and Pressman 2013). Despite considerable progress in the treatment of extensive skin defects, rapid and sufficient coverage remains a clinical challenge (Herndon and Spies 2001). Large defects are typically addressed using autologous split thickness skin grafts (STSG) (Andreassi et al. 2005; Lochbühler and Meuli 1992). In cases of massive burns, the availability of STSG may be insufficient. To address this scarcity, allogeneic donor skin, also known as cadaver skin, is often utilized for temporary wound coverage (Herndon and Spies 2001; Rowan et al. 2015). Early excision of burned tissue and temporary cadaver skin covering has reduced severe burn mortality (Ong et al. 2006). Donor skin does not usually integrate into the patient’s skin due to rejection reactions, instead it serves as a form of temporary wound covering (Richters et al. 2005).

Allografts are generally defined as transplants from a donor of the same species as the recipient (Andreassi et al. 2005). Skin allografts have been an integral component of in burn wound management for over a century, with their use dating back to 1881 (Girdner 1881). Concurrently, surgeons were experimenting with remnants from other surgical interventions such as amputations and circumcisions as allogeneic skin grafts (Klasen 2004; Lucas 1884).

From the 1970s onward, the supply of human allografts improved significantly in terms of standardized quality and biomedical safety with the development of skin banking facilities in HIC (Hoekstra et al. 1994). In some countries, the use of cadaver skin is prohibited due to religious or ethical principles, or it is unavailable due to a lack of infrastructure for skin banks. Therefore, allogenous skin grafts must often be donated from living close relatives (Lundy et al. 2011; Coruh et al. 2005). Alternatively, skin allografts can be sourced from the remains of plastic surgery procedures (Zidan and Eleowa 2014). These are usually employed immediately after donation and, when obtained from close relative, these grafts exhibit delayed and less pronounced rejection reactions (Megahed et al. 2021). A particularly intriguing subset of allografting, albeit scarcely reported in the medical literature, is grafting of dermal tissue between genetically identical, monozygotic twins. Here the grafting procedure may be considered autologous, given the genetic identity of the donor and recipient (Turk et al. 2014; Zhuang et al. 2022; Caruso et al. 1996; Ahmed et al. 2020).

Cryopreservation in liquid nitrogen (cryopreserved allografts—CPA) is a common method for preparing cadaver skin allografts for clinical use. Skin grafts are incubated with antibiotic solutions to prevent bacterial transmission (Pianigiani et al. 2006; Hermans 2011). CPA are frozen with dimethylsulfoxide (DMSO) as a cryoprotectant (Bravo et al. 2000). Some skin banks augment this method with radiation to decrease microbiological risks (Rooney et al. 2008). Alternatively, glycerol-preserved allografts (GPA) are prepared by glycerolizing samples in an ascending series of glycerol concentrations (Hermans 2011). To ensure bacteria neutralized, the samples are stored in glycerol at 2–8 °C for at least three weeks. Some suggest exposing the skin grafts to 98% glycerol (Marshall et al. 1995). The debate on how the preparation method affects immunogenicity and the speed of rejection continues. CPA typically retain more viable cells after preparation compared to GPA (Kua et al. 2012). It is assumed that living cells in the graft may provide growth and healing signals to the wound bed (Aggarwal et al. 1985). However, a literature review found that the available literature does not support this hypothesis (Hermans 2011). Clinical observational studies suggest the host immune system rejects CPA and GPA in a comparable time (Fletcher et al. 2013; Khoo et al. 2010). In a porcine wound model however, GPA exhibited fewer early inflammatory signs than CPA (Yoon et al. 2016). An advantage of GPA is its antibacterial and virucidal effects due to glycerolization, which diminishes the potential risk of transmission of viral agents, such as the human immunodeficiency virus (HIV) (Clarke 1987).

There are several indications for the use of allografts in the context of severe thermal injuries (Khoo et al. 2010). Their use as a temporary biological dressing in deep second- and third-degree burns helps restore the skin’s lost protective function, preventing loss of fluids, proteins, and electrolytes (Wang et al. 2020; Paggiaro et al. 2019). Additionally, allografts serve as a barrier against bacterial pathogens and reduce post-application pain (Khoo et al. 2010), bridging the gap until the patient’s condition allows autografting and until enough autografts are available. This intervention enhances vascularity, angiogenesis, and supports wound healing via cytokine and growth factor release (Khoo et al. 2010). Subsequently wounds are covered with autologous STSG (Herndon and Spies 2001).

In superficial partial thickness burns, allografts can serve as biological dressing eliminating the need for surgical intervention, preventing secondary progression of wound depth, often caused by desiccation or infection (Vloemans et al. 2003). Autologous skin grafts can be combined with allografts in a technique known as sandwich grafting. Allograft coverage safeguards the delicate autografts and the uncovered surfaces of the wound bed (Wang et al. 2020). Clinical data suggest that the slower and weaker rejection of GPA-based sandwich grafts may increase the probability of a successful wound closure (Kreis et al. 1989).

The aim of this review is to provide a comprehensive summary of current research and clinical practice concerning the use of allogeneic skin grafts in pediatric burns.

## Methods

**Fig. 1:**
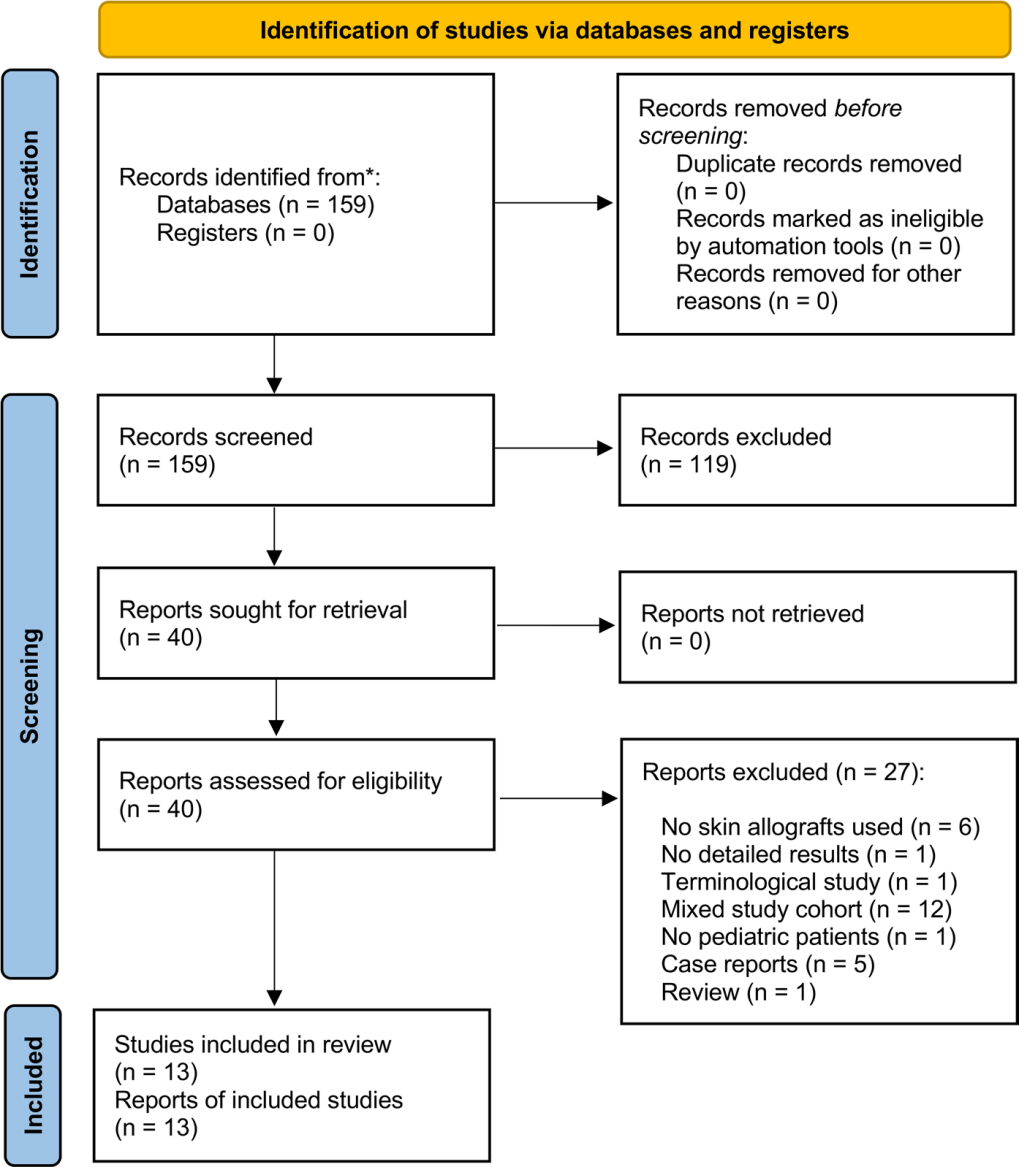
PRISMA 2020 flow diagram. A total of 159 publications were identified through systematic database search. After screening process, 13 studies were included for systematic review

This systematic review was preregistered at PROSPERO (number: CRD42024560654). A structured search strategy was conducted to identify relevant publications. The search was carried out in the medical data base PubMed using a combination of keywords related to thermal injuries, skin allograft treatment, and pediatric study populations, following the criteria of population, intervention, comparison, outcome (PICO). See Supplement 1 for the search strategy for PubMed. References in English or German, published between 01/2000 and 07/2024, were included. Studies with mixed cohorts containing both pediatric and adult burn patients, where separate analysis was not performed, were excluded. Only original articles reporting on randomized controlled studies (RCTs), or retrospective studies were included (Supplement 2). The selection process adhered to the Preferred Reporting Items for Systematic Reviews and Meta-Analyses Statement 2020 (PRISMA 2020) criteria (Page et al. 2020) (Fig. 1). For the PRISMA 2020 checklist and the PRISMA 2020 for abstract checklist see Supplement 3 and 4. Two independent reviewers screened the results, in the event of a divergent decision, a third independent investigator was consulted. Main outcome parameters were the rationales for the use, the types of allografts used, the global distribution of use and the safety of allografts (adverse events, take rate, infection rate). Population characteristics of allograft cohort and long-term effects (scar contractures, necessity for operative revisions, skin quality, aesthetic and functional outcome) were extracted to characterize the heterogeneity of study results. A risk of bias analysis for unrandomized study were performed using the methodological index for non-randomized studies (MINORS), see Supplement 5 (Slim et al. 2003). Non-randomized case control studies were assessed using the Newcastle–Ottawa Quality Assessment Scale (Supplement 6) (Ottawa Hospital Research Institute n.d 2024). For randomized studies the Cochrane risk-of-bias tool was used (Supplement 7) (Sterne et al. 2019).

## Results

The initial search query yielded a total of 159 studies. Subsequent screening, which involved reviewing the title and abstract, led to the identification of 40 studies, deemed suitable for comprehensive full-text assessment. Among these, a total of 13 studies met the criteria for qualitative analysis. Reasons for excluding the remaining studies are detailed in the PRISMA 2020 flow chart (Fig. 1).

**Fig. 2:**
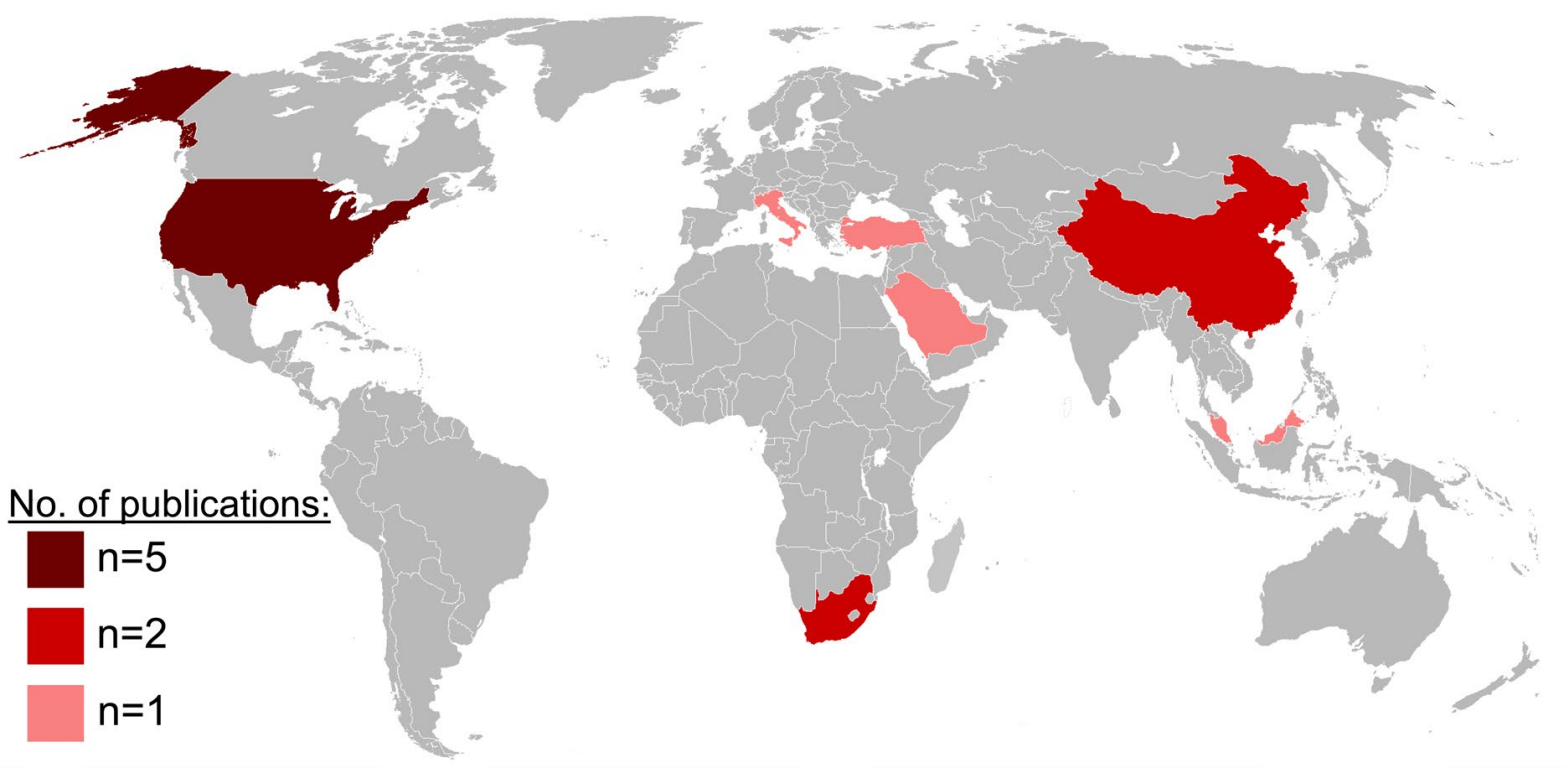
Geographical distribution of included studies. World map displaying the origin of all studies included in this systematic review. 58% (n = 7) were conducted in HIC with a clear predominance of studies from the US (n = 5). Further studies were conducted in Italy, Saudi Arabia, Turkey, Malaysia (all n = 1), China and South Africa (both n = 2)

Included studies were subject to systematic analysis, see Table 1 and Supplement 2. Among these, 11 studies were retrospective in nature, while 2 were RCTs. In accordance with the most recent classification system established by the World Bank Group (2023), 54% (n = 7) of the studies were conducted in HIC, while the remaining 46% (n = 6) were conducted in LMIC, for global distribution see Fig. 2.

**Table 1:** Table summarizing the included studies including characterization of allograft use, injury characteristics, postoperative dressing regimes, and origin of the studies

Study	Indication for use	Type of allograft	Allograft meshed?	Dressing regime	TBSA	Burn sites	Origin
Bosco et al. (2011) Martens et al. (2023)	Temporary wound coverage	CPA	Not mentioned	Not mentioned	11% (mean)	Hand, face, torso	HIC
Branski et al. (2007) Bosco et al. (2011)	Sandwich grafting technique	Not mentioned	Yes	Not mentioned	74% (mean)	Not specified	HIC
Coruh et al. (2005)	Sandwich grafting technique	Freshly donated allografts	Yes	Antiseptic tulle gras dressing	40–60% (range)	Not specified	LMIC
Cox et al. (2011) Stubbs et al. (2011)	Temporary wound coverage	Not mentioned	Not mentioned	Not mentioned	11.5% (mean)	Not specified	LMIC
Greenhalgh et al. (2013) Cuono et al. (1986)	Wound bed preparation	CPA	No	No postoperative dressing	39.5% (mean)	Face	HIC
Khoo et al. (2010)	Wound bed preparation	GPA	No	Paraffin or lipocoloid dressing	Not mentioned	Not specified	LMIC
Martens et al. (2023) Shen et al. (2021)	Temporary wound coverage	Not mentioned	Not mentioned	Not mentioned	66% (mean)	Not specified	HIC
Naoum et al. (2004) Zhao et al. (2023)	Temporary wound coverage	CPA, freshly donated allografts	Yes	Daily dressing changes	64% (mean)	Not specified	HIC
Puyana et al. (2019) Cuono et al. (1987)	Temporary wound coverage	Not mentioned	No	Moisture-retaining dressings	6.7% (mean)	Face	HIC
Qaryoute et al. (2001) Rimdeika and Bagdonas (2005)	Sandwich grafting technique	Freshly donated allografts	Yes	Not mentioned	50–65% (range)	Not specified	HIC
Rode et al. (2017) Qaryoute et al. (2001)	Wound bed preparation	Not mentioned	No	Polyamid gauze, bismuth-dressing	49.6% (mean)	Not specified	LMIC
Shen et al. (2021) Cox et al. (2011)	Temporary wound coverage	Freshly donated allografts	No	Mesh gauze, silver-dressing	31% (median)	Not specified	LMIC
Zhao et al. (2023) Rode et al. (2017)	Temporary wound coverage	Not mentioned	Not mentioned	First dressing changes on day 7	18.5% (mean)	Not specified	LMIC

### Effectiveness and safety of dermal allografts in pediatric burn care

Traditionally partial thickness burns were treated with daily debridement and the frequent application of silver sulfadiazine. A RCT by Naoum et al. compared this method with the application of allografts in partial thickness burns greater than 40% TBSA in a pediatric collective of 29 children (n = 16 in allograft group vs. n = 13 in sulfadiazine group). The allogeneic skin grafts were meshed and used as biological dressing. The two experimental groups were comparable regarding demographics and injury related data. Length of hospital stay (LOHS) was reduced in the allograft group (24 ± 9.6 vs. 40 ± 18.2 days, *p* = 0.008). The authors did not observe mortality in the allograft group, whereas treatment with sulfadiazine resulted in three fatal cases due to sepsis as a complication of a local infection (*p* = 0.08). The study established allografts as the gold standard for large-scale thermal injuries in pediatric patients (Naoum et al. 2004).

A RCT by Branski et al. compared the classic autograft-allograft technique (sandwich grafting technique), with the use of Integra dermal regeneration template (Integra LifeSciences, Princeton, NJ, USA) focusing on the clinical, aesthetic, and metabolic outcomes. The authors included 20 pediatric patients with burns > 50% TBSA. The percentage of predicted resting energy expenditure (PPREE) was used as an indicator for the metabolic status. The PPREE was elevated after 3 weeks and at discharge in the allograft group, whereas the Integra group had already normalized the metabolic aberrations. The acute phase reaction was greater when Integra was used. After 24 months the Integra group had an increased bone mineral content and density. Aesthetic outcome of the allograft group deemed inferior to that of the other patients, measured using the Hamilton burn scar scoring system (12 months: Integra 5.4 ± 1.7 vs. allograft 7.7 ± 2.6, *p* = 0.003; 24 months: Integra 4.3 ± 2.2 vs. allograft 6.6 ± 3.1, *p* = 0.02 [mean ± SD]). No significant differences in mortality rate were observed (30% in allograft group vs. 40% in Integra group). The authors conclude that Integra has a positive influence on early metabolic state and on long-term aesthetic outcomes. In both groups 4 cases of wound infections were reported with no differences regarding causative organisms (Branski et al. 2007).

Zhao compared the established allograft technique with a bacterial cellulose dressing (BCD) after severe scald injuries with at least 10% TBSA in 317 pediatric patients. All underwent eschar dermabrasion surgery and wound coverage by allografts or BCD. Surgery time was longer in BCD group (BCD 44.3 ± 7.0 min vs. allograft 31.5 ± 6.1 min [mean ± SD], *p* = 0.009). But the healing time was significantly shorter (BCD 19.6 ± 2.2 days vs. allograft 24.4 ± 4.3 days [mean ± SD], *p* = 0.002) and the number of dressing changes lower (BDS 3.5 ± 0.8 changes vs. allograft 6.7 ± 2.1 changes [mean ± SD], *p* = 0.006) in BCD group. 22.9% of all allograft cases needed further autografting, compared to 8.62% in BCD group (*p* = 0.036). No information regarding infection rates were provided. The authors conclude that BDC is a reliable substitute for allografts when available (Zhao et al. 2023).

A single-center retrospective study by Khoo et al. (2010) utilized GPA in 43 of 477 patients, including 19 patients below 12 years. Indications for the utilization of skin allografts (GPA) in this study encompassed wound bed preparation and the sandwich grafting technique for deep partial and full-thickness burns, and biological dressing for superficial partial-thickness burns. The authors claim a benefit from GPA wound bed preparation, improving the take rate of autografts. The study design does not allow any conclusions to be drawn regarding safety issues including wound infections (Khoo et al. 2010).

Bosco et al. conducted a retrospective analysis of the use of autologous skin in combination with allogeneic cryopreserved banked skin in 171 patients with burns and 4 patients with necrotizing fasciitis at the Burns Center of Verona. 29 out of 51 pediatric patients (< 15 years) with superficial partial thickness burns were treated with allografts, without the necessity of subsequent autologous skin grafting. Mean LOHS was 8.4 days. Additionally, 22 children were treated with CPA for wound bed preparation prior to autografting. Here mean LOHS was 20.0 days. Infection rates were not reported. At the 6 months follow-up no transmission of HIV, hepatitis C virus (HCV) or hepatitis B virus (HBV) was reported, and mortality was 0%, reflective of the relatively mild burn study population, with a mean TBSA of 16.0%. The absence of a control group limits comparative conclusions (Bosco et al. 2011).

Rode et al. used allografts in combination with STSG expanded by Meek procedures in 35 pediatric patients (age: 4.1 ± 3.08 years [mean ± SD], TBSA 49.5 ± 22.37% [mean ± SD]) in deep partial thickness or full thickness burns in South Africa. The allografts were either used as wound bed preparation prior to application of STSG, or in combination with STSG to achieve full skin cover. 8 patients (22.9%) died. The authors ascribe this high mortality rate to the extensive TBSA and the overall health of the patient cohort, including a high prevalence of chronic infectious conditions and malnutrition. Furthermore, they attribute the graft loss to wound infections, including one case of complete graft lost due to colonization by *Acinetobacter baumanii*. Wound infections were managed with sodium hypochlorite solutions and silver-containing dressings.

Among survivors, mean LOHS was 75.5 days. In 16 instances, the surgeons identified a need for allografts but faced a shortage of available skin. Long-term functional and aesthetic outcomes were deemed satisfactory in 5 patients at a 5-year follow-up (Rode et al. 2017).

Martens et al. evaluated their management algorithms for pediatric burn injuries > 50% TBSA, comparing the outcome of different wound dressings at the Shriners Hospital for Children Northern California, USA. The choice of initial dressing type—whether allograft, autograft, Integra, or xenograft—was not indicative of patient mortality. To minimize the need for multiple dressing changes, surgeons tended to avoid allografts in favor of alternative dressings, other important safety parameters such as infection rates were not assessed (Martens et al. 2023).

Qaryoute et al. reported on five cases of children with burns of 50–65% TBSA, in which fresh maternal allografts were utilized as the sandwich grafting technique in combination with meshed autografts, in Saudi-Arabia. The authors noted successful integration of both graft elements in all patients, with no evidence of significant rejection, although partial loss of the intermingled grafts occurred in some cases due to infection. Autografting was performed as a salvage procedure after graft loss. The authors performed punch biopsies in two cases, not showing any evidence of rejection. Mortality rate was 20%. According to the authors, freshly donated maternal allografts are a safe treatment option in patients with extensive TBSA (Qaryoute et al. 2001).

In a Turkish retrospective study by Coruh et al., the use of freshly donated allografts in a sandwich technique in combination with meshed autografts was assessed in 12 pediatric burn patients, TBSA being 52.0 ± 6.9% [mean ± SD], with a mortality rate of 50%. LOHS range was 44–130 days in surviving pediatric patients and 4–43 days in deceased pediatric patients. In cases of graft loss due to wound infections or insufficient initial wound debridement, repeated grafting was performed. Further conclusions regarding the outcomes of pediatric patients cannot be inferred since the study did not perform a separate subgroup analysis for this population. The authors note that using freshly donated allografts from close relatives minimizes the risk of viral transmission. They suggest a lower infection rate compared to GPA, but do not provide a direct comparison in their study (Coruh et al. 2005). Shen et al. utilized fresh allografts from the scalp of relatives for deep partial and full thickness burns. Donors underwent testing for HIV, HBV, HCV, and Treponema pallidum. 22 pediatric patients were included with a mean age of 3 years and 2.5 months, suffering from full-thickness burns exceeding 10% TBSA (range 10–86%). Mean LOHS was 57 days and there were no fatalities after 1 year. Infection rates were not documented (Shen et al. 2021).

Neonatal burn management poses unique challenges due to distinct anatomical and physiological traits (Rimdeika and Bagdonas 2005). The immaturity of the immune system demands complex intensive care management (Cox et al. 2011). Furthermore, neonates have an increased risk of long-term sequelae, such as hypertrophic scars and joint contractures (Burlinson et al. 2009). Cox et al. focused on burn victims below four months of age in Cape Town, South Africa with a mean TBSA of 11.5% (range 1–55%), primarily involving flame burn and scalds. Immediate autologous skin grafting was performed in 25 patients. In cases with extensive TBSA, allografts were used to achieve prompt full wound coverage. LOHS was longer in the allograft group in comparison to the immediate autograft group (45.2 vs. 30.8 days), yet the number of surgical procedures per patient (autograft: 1.6 (range 1–10) vs. allograft 1.3 (range 1–5)) and the average intraoperative blood requirement (autograft: 16.7 ml/kg (range 5-50 ml/kg) vs. allograft: 14.4 ml/kg (range 16-50 ml/kg)) was comparable among. The authors stress that prompt coverage using autografts or alternatively allografts is clearly superior to delayed grafting and reduces the rate of wound infections (Cox et al. 2011).

Two investigations explored the use of allografts for facial burn treatments in children. The face is crucial to a child's development, communication abilities, and self-esteem (Stubbs et al. 2011) and hypertrophic scarring can require a series of medical interventions to address disfiguring scars (Grevious et al. 2008). Greenhalgh et al. (2013) analyzed the use of allografts in pediatric facial burns, retrospectively. 28 out of 160 patients with facial burn were initially treated with CPA for the purpose of wound bed preparation before autografting; 105 patients were treated immediately with autografts, and 23 patients with Integra. Allogeneic grafts were used in high-risk patients (TBSA 69.9 ± 14.5% [mean ± SD]). The allograft and Integra groups had a higher rate of follow-up interventions. A higher rate of graft loss was observed in the Integra group, whereas the rate was comparable in the allograft und autograft groups.

Interestingly, there was no graft failure due to infection in the group which had initially undergone allografting, yet about 30% of all Integra grafts failed due to infection. However, the cadaver skin group and the integra group had significantly higher TBSA, limiting comparability. Furthermore, the informative value of the subgroup analysis of outcome parameters and complications is diminished due to the absence of statistical analysis. The authors conclude that allografts are an important part of the therapy of facial burns, in the context of a lack of autograft donor sites (Greenhalgh et al. 2013).

Puyana et al. examined the use of facial allografts in comparison to dehydrated human amniotic and chorionic membrane (DHACM) as a biological dressing, eliminating the need for further grafting. 30 patients under 16 years of age with partial thickness facial burns were selected for retrospective analysis. Both cohorts were statistically comparable regarding demographics and injury related characteristics. Full epithelialization was achieved in all patients by the second week post-surgery. At the one-year follow-up, one case of wound infection and three cases of hypertrophic scars were observed in the allograft group, whereas the DHACM group reported no complications (*p* = 0.045). The data suggest that DHACM may reduce the rate of complication in comparison to the use of cadaver skin for treating facial partial thickness burns (Puyana et al. 2019).

### A matter of choice and availability: CPA, GPA, or freshly donated allografts?

The choice and availability of allograft types—cryopreserved allografts (CPA), glycerol-preserved allografts (GPA), or freshly donated allografts—might make a significant difference on outcome. 2 out of 12 studies used CPA (Bosco et al. 2011; Greenhalgh et al. 2013), three used freshly donated allografts (Coruh et al. 2005; Qaryoute et al. 2001; Shen et al. 2021) and only one study used GPA (Khoo et al. 2010). One study combined CPA and freshly donated allografts (Naoum et al. 2004). In six studies the type of allograft used was not specified (Branski et al. 2007; Zhao et al. 2023; Rode et al. 2017; Martens et al. 2023; Cox et al. 2011; Puyana et al. 2019).

Khoo et al. (2010) used GPA in their retrospective study for wound bed preparation prior to autografting, or alternatively as a biological wound dressing without subsequent autografting in superficial partial thickness burns. The authors justified their choice on the premise that it is non-viable, which reduces its immunogenicity and enhances its microbiological safety when compared to CPA (Khoo et al. 2010).

Contrarily, Bosco et al. advocate for the use of cryopreserved allografts for superficial partial and full thickness burns. They perceive the preservation of cell viability following cryopreservation to be benefit over storage in glycerol, did however not perform comparative analysis (Bosco et al. 2011). Greenhalgh et al. also favored the use of frozen allografts for the treatment of facial burns, without however addressing the potential impact of their choice (Greenhalgh et al. 2013). Naoum et al. chose a combination of cryopreserved and freshly donated allografts, without elaborating on the rationale behind this selection (Naoum et al. 2004).

Besides CPA and GPA, the use of parental allografts is an alternative source of skin. Reasons for its application may be local ethical and religious beliefs that prevent tissue donation. Moreover, they are sometimes used due to their delayed rejection, in comparison to cadaver skin. Other methods to delay or even prevent allograft rejection included immunomodulation. As postulated by Qaryoute et al. (2001), close HLA matching might even allow permanent persistence of the parental graft, in the absence of immunomodulatory therapies. Although allografts are generally used as temporary wound coverage, rare cases of partial integration have been reported. In the 1980s, Cuono et al. demonstrated that the use of allogenic dermis as a scaffold for autografting could lead to permanent incorporation, likely facilitated by post-traumatic immunosuppression in severely burned patients. This phenomenon has been observed primarily with fresh or cryopreserved allografts, although processing methods such as radiation treatment may influence the likelihood of integration (Cuono et al. 1986, 1987).

In China Shen et al. (2021) and in Turkey Coruh et al. (2005) used fresh allografts donated by relatives, due to local ethical and religious beliefs. There is a scarcity of cadaver skin due to cultural and local governmental laws in China. Mean time to rejection of these allograft was 15.5 ± 3.60 days in the Chinese study. The authors mention that freshly donated skin from relatives shows a higher immune rejection compared to cadaver skin, possibly leading to a shorter time to rejection and thus the necessity of more frequent re-grafting, however they claim that re-harvesting from the same donor scalp was possible after an average of 7.6 ± 1.09 days and was performed twice in 11 donors and three times in 5 donors, with a low donor site morbidity (Shen et al. 2021).

Coruh et al. advocate for the use of freshly donated allografts from close relatives due to cultural resistance to skin donation and the absence of adequate facilities for skin processing. The authors elucidate their 'sandwich technique'—wherein skin autografts and allografts are used in conjunction. This technique facilitates epithelialization by the autograft's keratinocytes, occurring concurrently with the rejection of the allograft. Nevertheless, early partial loss of the intermingled allo- and autografts was observed in some cases, primarily due to infections or insufficient debridement. The authors propose that freshly donated allografts provide multiple advantages: allografts closely matched in HLA type show a longer lifespan and may show improved integration due to their maintained viability in compared to cadaver skin. Yet, the paper does not empirically confirm these hypotheses, and a comparative application or detailed examination is not provided (Coruh et al. 2005). All three studies using freshly donated allografts addressed the risk of transmission of infectious diseases by conducting preoperative testing for HIV and hepatitis, Shen et al. additionally tested for *Treponema pallidum* (Coruh et al. 2005; Qaryoute et al. 2001; Shen et al. 2021).

### The use of allografts and cadaver skin in LMIC

While skin banks in HIC generally ensure the availability of allogeneic skin graft products, maintaining standards of quality, safety, and reliability (Bosco et al. 2011; Vloemans et al. 2002), surgeons in other regions consistently encounter a shortage of allogeneic grafts (Roberson et al. 2020). This scarcity is further exacerbated by the constraints of societal, cultural, and political obstacles, coupled with an absence of both skin banks and legislation supporting skin donation. In the face of these issues, South Africa has emerged as a leading example in the field, by its launch of a cadaver skin bank in 2016 (Allorto et al. 2016; Rogers et al. 2013; Gupta et al. 2019). In the 1970s, the scarcity of allografts prompted Indian burn specialists to recognize the urgent need for skin banks. In 2000, Madhuri Gore, burn surgeon at Sion Hospital, Mumbai, India established a skin bank in collaboration with the Euro Skin Bank and Rotary International. The initiative was successful in harvesting skin from in-hospital deaths as well as networking with social groups, surmounting cultural and psychological obstacles and fostering a willingness to donate skin (Gore and De 2010; Gore 2017). Presently, the nation hosts 27 such banks, adhering to the stringent benchmarks set by the Euro Skin Bank. In 2015 the National Burn Center harvested 402,462cm^2^ donor skin from 261 deceased (Keswani et al. 2018). The skin bank of Choithram Hospital, Indore, India performed over 290 cadaver harvests since 2011 (Hospital and in Indore | Choithram Hospital n.d. 2024).

## Discussion

Early debridement of deep partial thickness or full thickness burns, followed by coverage with autografts, allografts or skin substitutes outperforms delayed excision and conservative therapy (Herndon and Spies 2001). Allogeneic skin grafts act as an interim measure, providing protection against wound bed infection and assisting in fluid loss control, helping to maintain hemodynamic homeostasis (Vigani and Culler 2017).

Several indications for the use of allogeneic skin grafts are documented. For superficial partial thickness burns, allografts serve as biological dressing, creating a conductive environment for epithelialization, promoting vascularization, and alleviating pain (Cleland et al. 2014). Applying allografts to such burns helps prevent the burn from progression to a more severe injury (Bosco et al. 2011). Moreover, allografts can serve as a temporary solution for wound bed preparation or as coverage for meshed autografts in the sandwich grafting technique (Khoo et al. 2010; Branski et al. 2007; Rode et al. 2017; Qaryoute et al. 2001).

The aim of this review was to determine the value of cadaver skin and freshly donated allografts in pediatric burn care. The search strategy yielded 13 studies for an in-depth analysis of the effectiveness and safety of dermal allografts. However, only 2 out of 13 studies being RCTs and risk of bias analysis revealed relevant limitations, the evidence level is restricted.

Study origin is critical due to healthcare disparities and graft availability. Worldwide, access to allografts varies: In Europe and North America, the provision of allografts is secured by skin banks (Hoekstra et al. 1994; Branski et al. 2007; Bosco et al. 2011; Greenhalgh et al. 2013). The Euro Skin Bank located in Beverwijk, The Netherlands, prepares over 1,000,000cm^2^ of cadaver skin annually for burn surgery within the European Union (Mackie 1997). While there have been efforts to set up similar institutions in LMIC, the demand far exceeds the supply (Roberson et al. 2020; Wall et al. 2018). Structural shortage is linked to avoidable mortality in burn patients (Allorto and Clarke 2015).

In regions where skin banks are unavailable or their use is limited by ethical and religious beliefs, freshly donated allogeneic skin grafts may offer a solution (Coruh et al. 2005; Qaryoute et al. 2001; Shen et al. 2021). HIC seldom use fresh allografts due to an ample supply of preserved cadaver skin and ethical concerns. Fresh allografts enhance vascularization and reduce infections. However, the risk of transmitting infections remains a concern, making preoperative donor screening essential. In this context, observations and research conducted in LMIC are indispensable. Since the majority of burn injuries worldwide occur in these regions, greater financial resources should be allocated to support local research and infrastructure development.

This review has limitations. Heterogeneity in follow-up protocols across studies resulted in limited comparability, with varying outcome parameters. A lack of systematic assessment of long-term outcomes, especially aesthetic, functional, and infectious parameters, limits robust conclusions. Some studies relied on subjective evaluations or lacked standardized objective measures. Future research should prioritize well-structured study designs with uniform follow-up protocols and validated objective tools and scoring systems. Lastly, several RCTs were omitted because they included mixed cohorts of adults and children without separate data analysis. Given the unique considerations in pediatric burn care, extrapolating results from mixed populations can be precarious.

## Conclusion

The studies surveyed in this review encompass patients who have been treated with diverse allografts for multiple clinical reasons. The effectiveness of these treatments is closely correlated with the extent and severity of the underlying burn injury. Scientific literature reports on the use of allografts are globally scant, which is noteworthy, given that most centers for severely burned children, at least in HIC, have incorporated allografts into their standard of care. To definitively determine the efficacy and outcomes associated with allograft employment, there is an exigent requirement for additional investigative studies, particularly for rigorously structured randomized controlled trials (RCTs). The advancement of burn medicine can be truly achieved only when the benchmarks are deeply and accurately comprehended.

## Supplementary Information

Below is the link to the electronic supplementary material.

Supplementary file1 (PDF 334 kb)

## Data Availability

No datasets were generated or analysed during the current study.

## Abbreviations

CPA: Cryopreserved allograft
DHACM: Dehydrated human amniotic and chorionic membrane
DMSO: Dimethyl sulfoxide—(CH_3_)_2_SO
FTSG: Full thickness skin graft
GPA: Glycerol-preserved allograft
HBV: Hepatitis B virus
HCV: Hepatitis C virus
HIC: High-income countries
HIV: Human immunodeficiency virus
LMIC: Low- and middle-income countries
LOHS: Length of hospital stay
PICO: Population, intervention, comparison, outcome
PPREE: Percentage of predicted resting energy expenditure
PRISMA: Preferred reporting items for systematic reviews and meta-analyses
RCT: Randomized controlled trial
STSG: Split thickness skin graft
TBSA: Total body surface area

## References

[CR1] Abarca L, Guilabert P, Martin N, Usúa G, Barret JP, Colomina MJ (2023) Epidemiology and mortality in patients hospitalized for burns in Catalonia. Spain Sci Rep 13:14364. 10.1038/S41598-023-40198-2 37658072 10.1038/s41598-023-40198-2 PMC10474035

[CR2] Aggarwal SJ, Baxter CR, Diller KR (1985) Cryopreservation of skin: an assessment of current clinical applicability. J Burn Care Rehabil 6:469–476. 10.1097/00004630-198511000-00003 3916431 10.1097/00004630-198511000-00003

[CR3] Ahmed R, Giwa L, Jordan N, Dheansa B (2020) The helpful twin: skin graft donation in a challenging burn case. JPRAS Open 27:58–62. 10.1016/J.JPRA.2020.11.014 33335965 10.1016/j.jpra.2020.11.014 PMC7732964

[CR4] Allorto NL, Clarke DL (2015) Merits and challenges in the development of a dedicated burn service at a regional hospital in South Africa. Burns 41:454–461. 10.1016/J.BURNS.2014.07.021 25149190 10.1016/j.burns.2014.07.021

[CR5] Allorto N, Rogers AD, Rode H (2016) Getting under our skin: introducing banked allograft skin to burn surgery in South Africa. S Afr Med J 106:865–866. 10.7196/SAMJ.2016.V106I9.10852 27601105 10.7196/SAMJ.2016.v106i9.10852

[CR6] Andreassi A, Bilenchi R, Biagioli M, D’Aniello C (2005) Classification and pathophysiology of skin grafts. Clin Dermatol 23:332–337. 10.1016/j.clindermatol.2004.07.024 16023927 10.1016/j.clindermatol.2004.07.024

[CR7] Armstrong M, Wheeler KK, Shi J, Thakkar RK, Fabia RB, Groner JI et al (2021) Epidemiology and trend of US pediatric burn hospitalizations, 2003–2016. Burns 47:551–559. 10.1016/J.BURNS.2020.05.021 33781634 10.1016/j.burns.2020.05.021

[CR8] Bosco F, Governa M, Rossati L, Vigato E, Vassanelli A, Aprili G et al (2011) The use of banked skin in the burns centre of Verona. Blood Transfus 9:156–161. 10.2450/2011.0107-09 21251463 10.2450/2011.0107-09 PMC3096858

[CR9] Branski LK, Herndon DN, Pereira C, Mlcak RP, Celis MM, Lee JO et al (2007) Longitudinal assessment of Integra in primary burn management: a randomized pediatric clinical trial. Crit Care Med 35:2615–2623. 10.1097/01.Ccm.0000285991.36698.E2 17828040 10.1097/01.CCM.0000285991.36698.E2

[CR10] Bravo D, Rigley TH, Gibran N, Strong DM, Newman-Gage H (2000) Effect of storage and preservation methods on viability in transplantable human skin allografts. Burns 26:367–378. 10.1016/S0305-4179(99)00169-2 10751705 10.1016/s0305-4179(99)00169-2

[CR11] Burlinson CEG, Wood FM, Rea SM (2009) Patterns of burn injury in the preambulatory infant. Burns 35:118–122. 10.1016/j.burns.2008.02.005 18947932 10.1016/j.burns.2008.02.005

[CR12] Caruso DM, Gregory MW, Schiller WR (1996) The use of skin from a monozygotic twin combined with cultured epithelial autografts as coverage for a large surface area burn: a case report and review of the literature. J Burn Care Rehabil 17:432–434. 10.1097/00004630-199609000-00011 8889868 10.1097/00004630-199609000-00011

[CR13] Clarke JA (1987) HIV transmission and skin grafts. Lancet 1:983. 10.1016/s0140-6736(87)90335-7 2882378 10.1016/s0140-6736(87)90335-7

[CR14] Cleland H, Wasiak J, Dobson H, Paul M, Pratt G, Paul E et al (2014) Clinical application and viability of cryopreserved cadaveric skin allografts in severe burn: a retrospective analysis. Burns 40:61–66. 10.1016/j.burns.2013.05.006 24018216 10.1016/j.burns.2013.05.006

[CR15] Coruh A, Tosun Z, Ozbebit U (2005) Close relative intermingled skin allograft and autograft use in the treatment of major burns in adults and children. J Burn Care Rehabil 26:471–477. 10.1097/01.bcr.0000185114.59640.b4 16278560 10.1097/01.bcr.0000185114.59640.b4

[CR16] Cox SG, Rode H, Darani AN, Fitzpatrick-Swallow VL (2011) Thermal injury within the first 4 months of life. Burns 37:828–834. 10.1016/j.burns.2011.02.003 21397403 10.1016/j.burns.2011.02.003

[CR17] Cuono C, Langdon R, McGuire J (1986) Use of cultured epidermal autografts and dermal allografts as skin replacement after burn injury. Lancet 1:1123–1124. 10.1016/S0140-6736(86)91838-6 2422513 10.1016/s0140-6736(86)91838-6

[CR18] Cuono CB, Langdon R, Birchall N, Barttelbort S, McGuire J (1987) Composite autologous-allogeneic skin replacement: development and clinical application. Plast Reconstr Surg 80:626–635. 10.1097/00006534-198710000-00029 3310055 10.1097/00006534-198710000-00029

[CR19] Fletcher JL, Caterson EJ, Hale RG, Cancio LC, Renz EM, Chan RK (2013) Characterization of skin allograft use in thermal injury. J Burn Care Res 34:168–175. 10.1097/BCR.0b013e318270000f 23292585 10.1097/BCR.0b013e318270000f

[CR20] Girdner J (1881) Skin grafting with grafts from a dead subject. Med Record NY 20:119–120

[CR21] Gore M (2017) Cadaver skin donation and skin bank. Indian J Burns 25:3. 10.4103/IJB.IJB_31_17

[CR22] Gore MA, De AS (2010) Deceased donor skin allograft banking: response and utilization. Indian J Plast Surg 43:S114. 10.4103/0970-0358.70732 21321645 10.4103/0970-0358.70732 PMC3038389

[CR23] Greenhalgh DG, Hinchcliff K, Sen S, Palmieri TL (2013) A ten-year experience with pediatric face grafts. J Burn Care Res 34:576–584. 10.1097/BCR.0b013e3182a22ea5 23966114 10.1097/BCR.0b013e3182a22ea5

[CR24] Grevious MA, Paulius K, Gottlieb LJ (2008) Burn scar contractures of the pediatric neck. J Craniofac Surg 19:1010–1015. 10.1097/SCS.0b013e318175f468 18650723 10.1097/SCS.0b013e318175f468

[CR25] Gupta S, Mohapatra D, Chittoria R, Subbarayan E, Reddy S, Chavan V et al (2019) Human skin allograft: is it a viable option in management of Burn patients? J Cutan Aesthet Surg 12:132. 10.4103/JCAS.JCAS_83_18 31413483 10.4103/JCAS.JCAS_83_18 PMC6676815

[CR26] Hermans MHE (2011) Preservation methods of allografts and their (lack of) influence on clinical results in partial thickness burns. Burns 37:873–881. 10.1016/j.burns.2011.01.007 21353745 10.1016/j.burns.2011.01.007

[CR27] Herndon DN, Spies M (2001) Modern burn care. Semin Pediatr Surg 10:28–31. 10.1053/spsu.2001.19389 11172570 10.1053/spsu.2001.19389

[CR28] Hoekstra MJ, Kreis RW, du Pont JS (1994) History of the Euro skin bank: the innovation of preservation technologies. Burns 20:S43–S47. 10.1016/0305-4179(94)90089-2 8198743 10.1016/0305-4179(94)90089-2

[CR29] Best Burn Surgery Hospital in Indore | Choithram Hospital n.d. https://choithramhospital.com/departments/burn-surgery/ (accessed March 29, 2024).

[CR30] Johnson EL, Maguire S, Hollén LI, Nuttall D, Rea D, Kemp AM (2017) Agents, mechanisms and clinical features of non-scald burns in children: a prospective UK study. Burns 43:1218–1226. 10.1016/J.BURNS.2017.01.036 28645715 10.1016/j.burns.2017.01.036

[CR31] Keswani SM, Mishra MG, Karnik S, Dutta S, Mishra M, Panda S et al (2018) Skin banking at a regional burns centre-the way forward. Burns 44:870–876. 10.1016/J.BURNS.2017.11.010 29661552 10.1016/j.burns.2017.11.010

[CR32] Khoo TL, Halim AS, Saad AZ, Dorai AA (2010) The application of glycerol-preserved skin allograft in the treatment of burn injuries: an analysis based on indications. Burns 36:897–904. 10.1016/j.burns.2009.03.007 20299154 10.1016/j.burns.2009.03.007

[CR33] Klasen HJ. History of Burns. Erasmus Pub; 2004.

[CR34] Kreis RW, Vloemans AF, Hoekstra MJ, Mackie DP, Hermans RP (1989) The use of non-viable glycerol-preserved cadaver skin combined with widely expanded autografts in the treatment of extensive third-degree burns. J Trauma Injury Infect Crit Care 29:51–54. 10.1097/00005373-198901000-00010 10.1097/00005373-198901000-00010 2642973

[CR35] Kua E, Goh C, Ting Y, Chua A, Song C (2012) Comparing the use of glycerol preserved and cryopreserved allogenic skin for the treatment of severe burns: differences in clinical outcomes and in vitro tissue viability. Cell Tissue Bank 13:269–279. 10.1007/s10561-011-9254-4 21484230 10.1007/s10561-011-9254-4

[CR36] Lochbühler H, Meuli M (1992) Current concepts in pediatric burn care: surgery of severe burns. Eur J Pediatr Surg 2:201–204. 10.1055/s-2008-1063440 1390545 10.1055/s-2008-1063440

[CR37] Lucas RC (1884) On prepuce grafting. Lancet 124:586–587. 10.1016/S0140-6736(02)13814-1

[CR38] Lundy JB, Cancio LC, King BT, Wolf SE, Renz EM, Blackbourne LH (2011) Experience with the use of close-relative allograft for the management of extensive thermal injury in local national casualties during operation Iraqi freedom. Am J Disaster Med 6:319–324. 10.5055/ajdm.2011.0071 22235604 10.5055/ajdm.2011.0071

[CR39] Mackie DP (1997) The Euro skin bank: development and application of glycerol-preserved allografts. J Burn Care Rehabil. 10.1097/00004630-199701001-00004 9063799 10.1097/00004630-199701001-00004

[CR40] Marshall L, Ghosh MM, Boyce SG, MacNeil S, Freedlander E, Kudesia G (1995) Effect of glycerol on intracellular virus survival: implications for the clinical use of glycerol-preserved cadaver skin. Burns 21:356–361. 10.1016/0305-4179(95)00006-2 7546258 10.1016/0305-4179(95)00006-2

[CR41] Martens S, Romanowksi K, Palmieri T, Greenhalgh D, Sen S (2023) Massive pediatric burn injury: a 10-year review. J Burn Care Res 44:670–674. 10.1093/JBCR/IRAB201 34718611 10.1093/jbcr/irab201

[CR42] Megahed MA, Elkashity SA, Talaab AA, AboShaban MS (2021) The impact of human skin allograft as a temporary substitute for early coverage of major burn wounds on clinical outcomes and mortality. Ann Burns Fire Disasters 34:67 34054389 PMC8126363

[CR43] Naoum JJ, Roehl KR, Wolf SE, Herndon DN (2004) The use of homograft compared to topical antimicrobial therapy in the treatment of second-degree burns of more than 40% total body surface area. Burns 30:548–551. 10.1016/j.burns.2004.01.030 15302419 10.1016/j.burns.2004.01.030

[CR44] Ong YS, Samuel M, Song C (2006) Meta-analysis of early excision of burns. Burns 32:145–150. 10.1016/j.burns.2005.09.005 16414197 10.1016/j.burns.2005.09.005

[CR45] Ottawa Hospital Research Institute n.d. https://www.ohri.ca/programs/clinical_epidemiology/oxford.asp (accessed July 11, 2024).

[CR46] Page MJ, McKenzie JE, Bossuyt PM, Boutron I, Hoffmann TC, Mulrow CD, The PRISMA et al (2020) Statement: an updated guideline for reporting systematic reviews. BMJ 2021:372. 10.1136/BMJ.N71 10.1136/bmj.n71 PMC8005924 33782057

[CR47] Paggiaro AO, Bastianelli R, Carvalho VF, Isaac C, Gemperli R (2019) Is allograft skin, the gold-standard for burn skin substitute? a systematic literature review and meta-analysis. J Plast Reconstr Aesthet Surg 72:1245–1253. 10.1016/j.bjps.2019.04.013 31176542 10.1016/j.bjps.2019.04.013

[CR48] Peck MD (2011) Epidemiology of burns throughout the world. Part I: distribution and risk factors. Burns 37:1087–1100. 10.1016/j.burns.2011.06.005 21802856 10.1016/j.burns.2011.06.005

[CR49] Peck M, Pressman MA (2013) The correlation between burn mortality rates from fire and flame and economic status of countries. Burns 39:1054–1059. 10.1016/j.burns.2013.04.010 23768720 10.1016/j.burns.2013.04.010

[CR50] Pianigiani E, Risulo M, Ierardi F, Sbano P, Andreassi L, Fimiani M et al (2006) Prevalence of skin allograft discards as a result of serological and molecular microbiological screening in a regional skin bank in Italy. Burns 32:348–351. 10.1016/j.burns.2005.10.005 16529868 10.1016/j.burns.2005.10.005

[CR51] Puyana S, Elkbuli A, Ruiz S, Bernal E, McKenney M, Lim R et al (2019) The use of dehydrated human amniotic/chorionic membrane skin substitute in the treatment of pediatric facial burn. J Craniofac Surg 30:2551–2554. 10.1097/scs.0000000000005826 31449203 10.1097/SCS.0000000000005826

[CR52] Qaryoute S, Mirdad I, Hamail AA (2001) Usage of autograft and allograft skin in treatment of burns in children. Burns 27:599–602. 10.1016/s0305-4179(00)00152-2 11525855 10.1016/s0305-4179(00)00152-2

[CR53] Richters CD, Hoekstra MJ, Du Pont JS, Kreis RW, Kamperdijk EWA (2005) Immunology of skin transplantation. Clin Dermatol 23:338–342. 10.1016/j.clindermatol.2004.07.022 16023928 10.1016/j.clindermatol.2004.07.022

[CR54] Rimdeika R, Bagdonas R (2005) Major full thickness skin burn injuries in premature neonate twins. Burns 31:76–84. 10.1016/j.burns.2004.04.009 15639370 10.1016/j.burns.2004.04.009

[CR55] Roberson JL, Pham J, Shen J, Stewart K, Hoyte-Williams PE, Mehta K et al (2020) Lessons learned from implementation and management of skin allograft banking programs in low- and middle-income countries: a systematic review. J Burn Care Res 41:1271–1278. 10.1093/JBCR/IRAA093 32504535 10.1093/jbcr/iraa093

[CR56] Rode H, Martinez R, Potgieter D, Adams S, Rogers AD (2017) Experience and outcomes of micrografting for major paediatric burns. Burns 43:1103–1110. 10.1016/j.burns.2017.02.008 28318749 10.1016/j.burns.2017.02.008

[CR57] Rogers AD, Allorto NL, Adams S, Adams KG, Hudson DA, Rode H (2013) Isn’t it time for a cadaver skin bank in South Africa? Ann Burns Fire Disasters 26:142 24563640 PMC3917142

[CR58] Rooney P, Eagle M, Hogg P, Lomas R, Kearney J (2008) Sterilisation of skin allograft with gamma irradiation. Burns 34:664–673. 10.1016/j.burns.2007.08.021 18226461 10.1016/j.burns.2007.08.021

[CR59] Rowan MP, Cancio LC, Elster EA, Burmeister DM, Rose LF, Natesan S et al (2015) Burn wound healing and treatment: review and advancements. Crit Care. 10.1186/s13054-015-0961-2 26067660 10.1186/s13054-015-0961-2 PMC4464872

[CR60] Shen C, Deng H, Sun T, Cai J, Li D, Li L et al (2021) Use of fresh scalp allografts from living relatives for extensive deep burns in children: a clinical study over 7 years. J Burn Care Res 42:323–330. 10.1093/jbcr/iraa155 32960969 10.1093/jbcr/iraa155

[CR61] Slim K, Nini E, Forestier D, Kwiatkowski F, Panis Y, Chipponi J (2003) Methodological index for non-randomized studies (minors): development and validation of a new instrument. ANZ J Surg 73:712–716. 10.1046/J.1445-2197.2003.02748.X 12956787 10.1046/j.1445-2197.2003.02748.x

[CR62] Sterne JAC, Savović J, Page MJ, Elbers RG, Blencowe NS, Boutron I et al (2019) RoB 2: a revised tool for assessing risk of bias in randomised trials. BMJ. 10.1136/bmj.l4898 31462531 10.1136/bmj.l4898

[CR63] Stubbs TK, James LE, Daugherty MB, Epperson K, Barajaz KA, Blakeney P et al (2011) Psychosocial impact of childhood face burns: a multicenter, prospective, longitudinal study of 390 children and adolescents. Burns 37:387–394. 10.1016/j.burns.2010.12.013 21330061 10.1016/j.burns.2010.12.013

[CR64] The World Bank Group. 2023 World Bank Country and Lending Groups 2023. https://datahelpdesk.worldbank.org/knowledgebase/articles/906519-world-bank-country-and-lending-groups

[CR65] Turk E, Karagulle E, Turan H, Oguz H, Abali ES, Ozcay N et al (2014) Successful skin homografting from an identical twin in a severely burned patient. J Burn Care Res. 10.1097/BCR.0B013E3182957572 23811789 10.1097/BCR.0b013e3182957572

[CR66] Vigani A, Culler CA (2017) Systemic and local management of Burn wounds. Vet Clin North America Small Animal Practice 47:1149–1163. 10.1016/j.cvsm.2017.06.003 10.1016/j.cvsm.2017.06.003 28802983

[CR67] Vloemans AF, Schreinemachers MC, Middelkoop E, Kreis RW (2002) The use of glycerol-preserved allografts in the Beverwijk Burn centre: a retrospective study. Burns 28(Suppl 1):S2-9. 10.1016/s0305-4179(02)00084-0 12237056 10.1016/s0305-4179(02)00084-0

[CR68] Vloemans AF, Soesman AM, Suijker M, Kreis RW, Middelkoop E (2003) A randomised clinical trial comparing a hydrocolloid-derived dressing and glycerol preserved allograft skin in the management of partial thickness burns. Burns 29:702–710. 10.1016/s0305-4179(03)00161-x 14556729 10.1016/s0305-4179(03)00161-x

[CR69] Wall S, Allorto N, Weale R, Kong V, Clarke D (2018) Ethics of Burn wound care in a low-middle income country. AMA J Ethics 20:575–580. 10.1001/JOURNALOFETHICS.2018.20.6.MSOC1-1806 29905136 10.1001/journalofethics.2018.20.6.msoc1-1806

[CR70] Wang C, Zhang F, Lineaweaver WC (2020) Clinical applications of allograft skin in burn care. Ann Plast Surg 84:S158–S160. 10.1097/SAP.0000000000002282 32028339 10.1097/SAP.0000000000002282

[CR71] Yoon C, Lim K, Lee S, Choi Y, Choi Y, Lee J (2016) Comparison between cryopreserved and glycerol-preserved allografts in a partial-thickness porcine wound model. Cell Tissue Bank 17:21–31. 10.1007/s10561-015-9521-x 26150190 10.1007/s10561-015-9521-x

[CR72] Zhao R, Zhao C, Zhang Y, Wan Y, Wang Y (2023) Retrospective comparison of postoperative dressing after eschar dermabrasion on paediatric scald wounds: bacterial cellulose dressing and allogenic skin. Int Wound J. 10.1111/IWJ.14492 37989716 10.1111/iwj.14492 PMC10898373

[CR73] Zhuang M, Wang W, Cui Q, Sun Y (2022) Successful coverage of extensive burns using only the scalp of an identical twin as donor with modified meek micrografting technique. Asian J Surg 45:788–791. 10.1016/J.ASJSUR.2021.12.027 34961723 10.1016/j.asjsur.2021.12.027

[CR74] Zidan SM, Eleowa SA (2014) Banking and use of glycerol preserved full-thickness skin allograft harvested from body contouring procedures. Burns 40:641–647. 10.1016/j.burns.2013.08.039 24070848 10.1016/j.burns.2013.08.039

