# Supplementary material for: Availability, effectiveness and safety of cadaveric and fresh allogeneic skin grafts in pediatric burn care—a review
Source: Cell Tissue Bank. 2025 Mar 15;26(2):16. doi: 10.1007/s10561-025-10161-8 (PMC11910407; doi:10.1007/s10561-025-10161-8)

Supplement 1: Search strategy for PubMed

Date of search: 15 July 2024

Publication date: January 2000 - July 2024

Language: English, German

#1	scald[Title/Abstract]
#2	“BURNS”[MESH Terms:noexp]
#3	burn*[Title/Abstract]
#4	#1 OR #2 OR #3
#5	Allotrans*[Title/Abstract]
#6	Allograf*[Title/Abstract]
#7	Allogen*[Title/Abstract]
#8	“cadaver skin”[Title/Abstract]
#9	“Allografts”[MESH Terms:noexp]
#10	“Transplantation, homologous”[MESH Terms]
#11	#5 OR #6 OR #7 OR #8 OR #9 OR 10
#12	Pediatri*[Title/Abstract]
#13	“Infant”[MESH Terms]
#14	Infant*[Title/Abstract]
#15	Infancy [Title/Abstract]
#16	Newbor*[Title/Abstract]
#17	Baby[Title/Abstract]
#18	Paediatri*[Title/Abstract]
#19	Neonat*[Title/Abstract]
#20	“Child”[MESH Terms]
#21	Child*[Title/Abstract]
#22	Kid[Title/Abstract]
#23	Kids[Title/Abstract]
#24	Toddler[Title/Abstract]
#25	“Adolescent”[MESH Terms]
#26	“Pediatrics”[MESH Terms]
#27	#12 OR #13 OR #14 OR #15 OR #16 OR #17 OR #18 OR #19 OR #20 OR #21 OR #22 OR #23 OR #24 OR #25 OR #26
#28	#4 AND #11 AND #27

Supplement 2: Table of all studies included

Author	Journal, Year	Title	Type of study (City, Country)	Aim	Population characteristics	Use of allografts	Outcome
**Bosco et al.**	Blood Transfusion, 2011	The use of banked skin in the Burns Centre of Verona	Single center retrospective study (Verona, Italy)	Assessment of the indications for the use of allografts, the mortality rate, the LOHS, and the risk for viral transmissions.	51 pediatric patients, <15 years, mean age 4.0 years. Mean TBSA 11% (superficial partial thickness burns) vs. 16% (full thickness burns). Types of injury: flame burns (43.7%), scalds (29.5%), contact burns (26.8%).	CPA on superficial partial thickness burns at the time of admission (n=29) to protect the remaining dermis and to avoid further deepening (no debridement). In full-thickness burns (n=22) as temporary coverage before definitive autografting.	LOHS (mean): Superficial partial thickness burns: 8.4 days. Full thickness burns: 20 days.<br>Mortality: 0/51 (0%).<br>No transmission of HIV, HCV, HBV (follow-up at 6 months). Currently the best wound dressing for thermal injuries regarding functional and aesthetic outcome and cost efficiency.
**Branski et al.**	Critical Care Medicine, 2007	Longitudinal assessment of Integra in primary burn management: a randomized pediatric clinical trial	Single center randomized controlled trial (Galveston, TX, USA)	Comparison of Integra to the established autograft-allograft technique in terms of clinical outcome, hypermetabolic response, long-term cosmetic and functional outcome up to 24 months post injury.	20 pediatric patients, mean age 7.4 years (Integra) vs. 6.2 years (allograft). Mean ± SD TBSA 70% ± 5% (Integra) vs. 74% ± 4% (allograft). Types of injury not mentioned.	Allograft group: expanded autograft (meshed 1:4) with allograft overlay on as much area as possible and allografts on remaining areas (meshed 1:1.5). Type of allograft preparation not mentioned.	LOHS (mean ± SD): 38.2 ± 11.1 days (allograft) vs. 32.6 ± 15.0 days (Integra).<br>Mortality: 3/10 in allografts (30%) vs. 4/10 in Integra group (40%).<br>Later normalization of the percentage of predicted resting energy expenditure (PPREE) in the allograft. Significantly increased bone mineral content and density after 24 months, acute phase response and cosmetic outcome in Integra group. No significant differences regarding cardiac function (heart rate, stroke volume, cardiac index, cardiac output), liver size, muscle fractional synthetic rate, sepsis scores, and long-term functional outcome.
**Coruh et al.**	Journal of Burn Care & Rehabilitation, 2005	Close Relative Intermingled Skin Allograft and Autograft Use in the Treatment of Major Burns in Adults and Children	Single center retrospective study (Kayseri, Turkey)	Analysis of safety and outcome after using skin allografts from a close relative, as an alternative in case of limited autograft donor sites.	12 pediatric patients, age range 2-16 years, TBSA range 40-60%. Types of injury: flame burns, scalds.	Transplantation of a thin autograft (0.3 mm), subsequently covered by a meshed fresh allograft, donated by close relatives after debridement.	LOHS (range): 44-130 days.<br>Mortality: 6/12 (50%).<br>Multiple skin grafting procedures in survivors because of an allograft rejection.
**Cox et al.**	Burns, 2011	Thermal injury within the first 4 months of life	Single center retrospective study (Cape Town, South Africa)	Reviewing the clinical management and the role of surgery of newborns and infants under 4 months admitted to a burn unit.	86 pediatric patients, age < 4 months. Mean TBSA 11.5% (range 1-55%). Types of injury: flame burns (38/86), scalds (45/86).	Surgery in all patients with deep partial thickness or full thickness burns (n=59). Early escharotomy with temporary cadaver allograft with delayed autografting (n=27). Immediate autografting (n=25). Type of allograft preparation not mentioned.	LOHS (average): 30.8 days (autograft) vs. 45.2 days (allograft).<br>Mortality: 8/86 (9.3%).<br>Comparable numbers of surgical procedures and surgical blood requirement, but no statistical analysis. Authors preferring immediate autografting in the case of sufficient donor sites.

Author	Journal, Year	Title	Type of study (City, Country)	Aim	Population characteristics	Use of allografts	Outcome
**Greenhalgh et al.**	Journal of Burn Care & Research, 2013	A ten-year experience with pediatric face grafts	Single center retrospective study (Sacramento, CA, USA)	Reviewing the aesthetic outcome and need for subsequent reconstruction after initial facial skin grafts in children.	160 pediatric patients with facial burns, mean age 5.8 years, range 0.2-17 years. Mean TBSA 39.4%, range 1-98%. Types of injury: flame burns, scalds, contact burns, electric burns.	CPA as cover for the facial wound after facial excision until enough autograft donor sites were available.	LOHS (mean ± SD): 72.1 ± 73.7 days Mortality: 22/160 (13.8%). Usage of allografts in 30 out of 158 first surgical procedures (tendency for usage in higher-risk patients (mean TBSA 69.9%)). 35.3% meshed. Patients with allografts: higher rate of reconstruction procedures (approx. 65 vs. 55%), higher rate of graft loss (approx. 30 vs. 25%). Higher rate of undesirable outcomes (39% mortality, 43% required regrafting) in allograft group. Possible confounder however: allograft group had more severe burns.
**Khoo et al.**	Burns, 2010	The application of glycerol-preserved skin allograft in the treatment of burn injuries: an analysis based on indications	Single center retrospective study (Kubang Kerian, Malaysia)	Analyze the experience with allografts for different indication in burn treatment.	19 pediatric patients (<12 years). Mean TBSA for pediatric patients not indicated. Types of injury: flame burn, scalds, chemical burns, electrical burns.	GPA in deep partial thickness/full thickness burns for wound bed preparation or Sandwich Grafting Technique. In superficial partial thickness burns as definitive dressing.	LOHS (mean ± SD): not differentiated for pediatric patients, in total 42.9 ± 20.2 days. Mortality: not differentiated for pediatric patients, in total 12/29 (41.4%) Rate of complications or outcomes not differentiated for pediatric patients. Increased hemodynamic instability and higher mortality and morbidity in children in comparison to adult burn patients.
**Martens et al.**	Journal of Burn Care & Research, 2023	Massive Pediatric Burn Injury: A 10-Year Review	Single center retrospective study (Sacramento, CA, USA)	Analyze the treatment strategies and outcomes of massive pediatric burns.	69 pediatric patients, mean age 8.7, range not mentioned. Mean ± SD TBSA 66% ± 12%. Types of injury not mentioned.	Application of allografts in 52% of all cases at first surgery. Type of allograft preparation not mentioned.	LOHS (mean ± SD): 90 ± 60 days. Mortality: 17/69 (24.6%) Type of coverage (allograft, autograft, xenograft, Integra) at first surgery was no predictor of mortality. Surgeons preferred to use alternative dressings because of the frequent need for replacement of allografts.
**Naoum et al.**	Burns, 2004	The use of homograft compared to topical antimicrobial therapy in the treatment of second-degree burns of more than 40% total body surface area	Single center randomized controlled trial (Galveston, TX, USA)	Comparing topical antimicrobial therapy with allografts for treatment of partial thickness burns >40% TBSA.	29 pediatric patients, mean ± SD age 7.1 ± 6.3 years. Mean ± SD TBSA 64% ± 14.7%. Types of injury not mentioned.	Partial thickness burns (n=16): Freshly donated and cryopreserved allografts meshed 2:1 for wound coverage after debridement. Control group (n=13): treated with daily debridement and application of silver sulfadiazine.	LOHS (mean ± SD): 24 ± 9.6 days in allograft vs. 40 ± 18.2 days in control group (p<0.008). Mortality: 0/16 (0%) in allograft vs. 3/13 (23.1%) in control group (p=0.08). Equal number of operations and blood transfusions. 3 operative revision due to burn scar contractures in topical treatment group (23,1%) vs. 2 in the allograft group (12,5%).
**Puyana et al.**	The Journal of Craniofacial Surgery, 2019	The Use of Dehydrated Human Amniotic/Chorionic Membrane Skin Substitute in the Treatment of Pediatric Facial Burn	Multicenter retrospective study (Miami and Tampa, FL, USA)	Comparing the outcomes of dehydrated human amniotic and chorionic membrane (DHACM) and allografts in pediatric population with facial burns.	30 pediatric patients with partial thickness facial burns (mean age 3.7 years, range 0-15 years). Mean TBSA 6.7% (range 2-27%). Types of injury not mentioned.	Allografts after early debridement as skin substitute. Type of allograft preparation not mentioned. Early cohort: Treatment with allografts. Late Cohort: Treatment with DHACM.	LOHS: not mentioned. Mortality: 0/30 (0%). Allograft group: higher complication rate after 12 months (3 hypertrophic scars, 1 wound infection). DHACM group: no complication.

Author	Journal, Year	Title	Type of study (City, Country)	Aim	Population characteristics	Use of allografts	Outcome
**Qaryoute et al.**	Burns, 2001	Usage of autograft and allograft skin in treatment of burns in children	Multicenter retrospective study (Dhahran and Taif, Saudi Arabia)	Analyzing the clinical outcome of 5 pediatric burn patients treated with close relative allografts.	5 pediatric patients (age range 5-13 years). Range of TBSA: 50-65%. Types of injury: scald (1/5), flame burns (4/5).	Freshly donated maternal allografts meshed 1.5:1 to 3:1 to cover widely expanded autografts (Sandwich Grafting Technique)	LOHS: not mentioned. Mortality: 1/5 (20%). With intact graft – cause of death not mentioned. Successful take of both layers of the graft. No rejection in exemplary biopsies. Further autografts in 2 patients.
**Rode et al.**	Burns, 2017	Experience and outcomes of micrografting for major pediatric burns	Single center retrospective study (Cape Town, South Africa)	Reviewing the experience with Meek procedure (in some cases in combination with allografting) in a pediatric burn center.	35 pediatric patients (mean age 4.4 years, range 0.25-11 years). Mean TBSA: 49.6% (range 15-86%). Types of injury: scald, flame burns.	For wound bed preparation or in addition with Meek procedure to achieve full skin cover. Type of allograft preparation not mentioned.	LOHS (mean): 75.5 days (survivors). Mortality: 8/35 (22.9%). Lower mean ABSI score in survivors (5.9 vs. 10.1). 21 patients received allografts. Allografts as wound bed preparation (n=3) to achieve a good take rate after micrografting (mortality: 0/3). Allografts to cover Meek grafts after surgery (n=5) to protect the graft and to support epithelization (mortality: 1/5). Allograft pre and post Meek procedure (n=11) (mortality: 5/11). No allografts available in 16 cases. Excellent long term functional and aesthetic outcomes (only 5 patients attended follow up consultations for 5 years).
**Shen et al.**	Journal of Burn Care & Research, 2021	Use of Fresh Scalp Allografts From Living Relatives for Extensive Deep Burns in Children: A Clinical Study Over 7 Years	Single center retrospective study (Beijing, China)	Evaluating an alternative treatment option for major pediatric burns when frozen allografts are unavailable.	22 pediatric patients (<12 years, median 3.2, range 0.75 -11.8). Median TBSA 31% (range 10-86%). Types of injury: flame burn (7/22), scalds (15/22)	Fresh, close relative allografts were used to cover deep partial thickness and full thickness burns.	LOHS (mean): 57 days. Mortality: 0/22 (0%). Allografts turned red on day 3. Begin of rejection: day 7 post grafting. Logarithmic relationship between TBSA and number of donors needed to cover all wounds (R^2=0.8374).
**Zhao et al.**	International Wound Journal, 2023	Retrospective comparison of postoperative dressing after eschar dermabrasion on paediatric scald wounds: Bacterial cellulose dressing and allogenic skin	Single center retrospective study (Jinan, China)	Comparing the outcomes of Bacterial Cellulose Dressing (BCD) and allografts in pediatric population with deep partial thickness burns.	317 pediatric patients, mean age 24.1 months (BCD) vs. 17.3 months (allograft). Mean ± SD TBSA 21.5% ± 7% (BCD) vs. 18.5% ± 4.8% (allograft). Types of injury not mentioned.	Allografts were used as wound coverage after eschar dermabrasion. Type of allograft preparation not mentioned.	LOHS (mean): not mentioned. Mortality: 0/317 (0%) Shorter surgery time, but higher number of dressing changes, longer healing time in allograft group compared to BCD.

Supplement 3: PRISMA 2020 Checklist

Section and Topic	Item #	Checklist item	Location where item is reported
**TITLE**			
Title	1	Identify the report as a systematic review.	Title, p. 1
**ABSTRACT**			
Abstract	2	See the PRISMA 2020 for Abstracts checklist.	Abstract, p. 2
**INTRODUCTION**			
Rationale	3	Describe the rationale for the review in the context of existing knowledge.	Introduction, p. 3
Objectives	4	Provide an explicit statement of the objective(s) or question(s) the review addresses.	Introduction, p. 4
**METHODS**			
Eligibility criteria	5	Specify the inclusion and exclusion criteria for the review and how studies were grouped for the syntheses.	Methods, p. 5
Information sources	6	Specify all databases, registers, websites, organisations, reference lists and other sources searched or consulted to identify studies. Specify the date when each source was last searched or consulted.	Methods, p. 5,supp. 1
Search strategy	7	Present the full search strategies for all databases, registers and websites, including any filters and limits used.	Supp. 1
Selection process	8	Specify the methods used to decide whether a study met the inclusion criteria of the review, including how many reviewers screened each record and each report retrieved, whether they worked independently, and if applicable, details of automation tools used in the process.	Methods, p. 5
Data collection process	9	Specify the methods used to collect data from reports, including how many reviewers collected data from each report, whether they worked independently, any processes for obtaining or confirming data from study investigators, and if applicable, details of automation tools used in the process.	Methods, p. 5
Data items	10a	List and define all outcomes for which data were sought. Specify whether all results that were compatible with each outcome domain in each study were sought (e.g. for all measures, time points, analyses), and if not, the methods used to decide which results to collect.	Methods, p. 5,supp. 2
	10b	List and define all other variables for which data were sought (e.g. participant and intervention characteristics, funding sources). Describe any assumptions made about any missing or unclear information.	Methods, p. 5, supp. 2
Study risk of bias assessment	11	Specify the methods used to assess risk of bias in the included studies, including details of the tool(s) used, how many reviewers assessed each study and whether they worked independently, and if applicable, details of automation tools used in the process.	Methods, p. 5
Effect measures	12	Specify for each outcome the effect measure(s) (e.g. risk ratio, mean difference) used in the synthesis or presentation of results.	Methods, p. 5, supp. 2
Synthesis methods	13a	Describe the processes used to decide which studies were eligible for each synthesis (e.g. tabulating the study intervention characteristics and comparing against the planned groups for each synthesis (item #5)).	Methods, p 5, ln. 152
	13b	Describe any methods required to prepare the data for presentation or synthesis, such as handling of missing summary statistics, or data conversions.	N/A
	13c	Describe any methods used to tabulate or visually display results of individual studies and syntheses.	Supp. 2

Section and Topic	Item #	Checklist item	Location where item is reported
	13d	Describe any methods used to synthesize results and provide a rationale for the choice(s). If meta-analysis was performed, describe the model(s), method(s) to identify the presence and extent of statistical heterogeneity, and software package(s) used.	Methods, p. 5
	13e	Describe any methods used to explore possible causes of heterogeneity among study results (e.g. subgroup analysis, meta-regression).	Methods, p. 5, ln. 132
	13f	Describe any sensitivity analyses conducted to assess robustness of the synthesized results.	N/A
Reporting bias assessment	14	Describe any methods used to assess risk of bias due to missing results in a synthesis (arising from reporting biases).	N/A
Certainty assessment	15	Describe any methods used to assess certainty (or confidence) in the body of evidence for an outcome.	N/A
**RESULTS**			
Study selection	16a	Describe the results of the search and selection process, from the number of records identified in the search to the number of studies included in the review, ideally using a flow diagram.	Results, p. 6, Fig. 1
	16b	Cite studies that might appear to meet the inclusion criteria, but which were excluded, and explain why they were excluded.	Fig. 1
Study characteristics	17	Cite each included study and present its characteristics.	References, p. 16-20, supp. 2
Risk of bias in studies	18	Present assessments of risk of bias for each included study.	Supp. 5-7
Results of individual studies	19	For all outcomes, present, for each study: (a) summary statistics for each group (where appropriate) and (b) an effect estimate and its precision (e.g. confidence/credible interval), ideally using structured tables or plots.	Supp. 2
Results of syntheses	20a	For each synthesis, briefly summarise the characteristics and risk of bias among contributing studies.	Supp. 2, 5-7
	20b	Present results of all statistical syntheses conducted. If meta-analysis was done, present for each the summary estimate and its precision (e.g. confidence/credible interval) and measures of statistical heterogeneity. If comparing groups, describe the direction of the effect.	N/A (no statistical synthesis conducted)
	20c	Present results of all investigations of possible causes of heterogeneity among study results.	N/A
	20d	Present results of all sensitivity analyses conducted to assess the robustness of the synthesized results.	N/A
Reporting biases	21	Present assessments of risk of bias due to missing results (arising from reporting biases) for each synthesis assessed.	N/A
Certainty of evidence	22	Present assessments of certainty (or confidence) in the body of evidence for each outcome assessed.	N/A
**DISCUSSION**			
Discussion	23a	Provide a general interpretation of the results in the context of other evidence.	Discussion, p. 13-14
	23b	Discuss any limitations of the evidence included in the review.	Discussion, p. 14,

Section and Topic	Item #	Checklist item	Location where item is reported
			ln. 414
	23c	Discuss any limitations of the review processes used.	Discussion, p. 14, ln. 414
	23d	Discuss implications of the results for practice, policy, and future research.	Discussion, p. 14, ln. 419
**OTHER INFORMATION**			
Registration and protocol	24a	Provide registration information for the review, including register name and registration number, or state that the review was not registered.	Methods, p. 6
	24b	Indicate where the review protocol can be accessed, or state that a protocol was not prepared.	Methods, p. 5
	24c	Describe and explain any amendments to information provided at registration or in the protocol.	N/A
Support	25	Describe sources of financial or non-financial support for the review, and the role of the funders or sponsors in the review.	Funding Source, p. 15
Competing interests	26	Declare any competing interests of review authors.	Conflict of Interest, p. 15
Availability of data, code and other materials	27	Report which of the following are publicly available and where they can be found: template data collection forms; data extracted from included studies; data used for all analyses; analytic code; any other materials used in the review.	Results, p. 6, supp. 2

From: Page MJ, McKenzie JE, Bossuyt PM, Boutron I, Hoffmann TC, Mulrow CD, et al. The PRISMA 2020 statement: an updated guideline for reporting systematic reviews. BMJ 2021;372:n71. doi: 10.1136/bmj.n71

Supplement 4: PRISMA 2020 for Abstracts Checklist

Section and Topic	Item #	Checklist item	Reported (Yes/No)
**TITLE**			
Title	1	Identify the report as a systematic review.	Yes
**BACKGROUND**			
Objectives	2	Provide an explicit statement of the main objective(s) or question(s) the review addresses.	Yes
**METHODS**			
Eligibility criteria	3	Specify the inclusion and exclusion criteria for the review.	Yes
Information sources	4	Specify the information sources (e.g. databases, registers) used to identify studies and the date when each was last searched.	Yes
Risk of bias	5	Specify the methods used to assess risk of bias in the included studies.	Yes
Synthesis of results	6	Specify the methods used to present and synthesise results.	Yes
**RESULTS**			
Included studies	7	Give the total number of included studies and participants and summarise relevant characteristics of studies.	Yes
Synthesis of results	8	Present results for main outcomes, preferably indicating the number of included studies and participants for each. If meta-analysis was done, report the summary estimate and confidence/credible interval. If comparing groups, indicate the direction of the effect (i.e. which group is favoured).	Yes
**DISCUSSION**			
Limitations of evidence	9	Provide a brief summary of the limitations of the evidence included in the review (e.g. study risk of bias, inconsistency and imprecision).	Yes
Interpretation	10	Provide a general interpretation of the results and important implications.	Yes
**OTHER**			
Funding	11	Specify the primary source of funding for the review.	Yes
Registration	12	Provide the register name and registration number.	Yes

From: Page MJ, McKenzie JE, Bossuyt PM, Boutron I, Hoffmann TC, Mulrow CD, et al. The PRISMA 2020 statement: an updated guideline for reporting systematic reviews. BMJ 2021;372:n71. doi: 10.1136/bmj.n71

Supplement 5: Methodological index for non-randomized studies (MINORS) instrument. Items are scored as 0 (not reported), 1 (reported but inadequate), or 2 (reported and adequate). The maximum score for non-comparative studies is 16, and for comparative studies is 24.

Author (year)	#1	#2	#3	#4	#5	#6	#7	#8	#9	#10	#11	#12	score	risk of bias
**Bosco et al. (2011)**	2	2	1	2	0	2	0	0	N/A	N/A	N/A	N/A	09/16	moderate
**Coruh et al. (2005)**	2	2	0	1	0	0	0	0	N/A	N/A	N/A	N/A	05/16	high
**Cox et al. (2011)**	2	2	0	2	0	0	0	0	N/A	N/A	N/A	N/A	06/16	high
**Greenhalgh et al. (2013)**	2	2	2	2	0	2	0	0	N/A	N/A	N/A	N/A	10/16	moderate
**Khoo et al. (2010)**	2	2	2	2	0	0	2	0	N/A	N/A	N/A	N/A	10/16	moderate
**Martens et al. (2023)**	2	2	2	2	0	2	0	0	N/A	N/A	N/A	N/A	10/16	moderate
**Puyana et al. (2019)**	2	2	2	2	0	2	1	0	2	0	2	2	17/24	moderate
**Qaryoute et al. (2001)**	2	2	0	2	0	1	0	0	N/A	N/A	N/A	N/A	07/16	high
**Rode et al. (2017)**	2	2	2	2	0	2	2	0	N/A	N/A	N/A	N/A	12/16	moderate
**Shen et al. (2021)**	2	2	1	2	0	2	1	0	N/A	N/A	N/A	N/A	10/16	moderate
**Zhao et al. (2023)**	2	2	1	2	1	2	0	0	2	2	2	2	18/24	moderate

Interpretation:

MINORS-Score (non-comparative studies)

<8	high risk of bias
8-12	moderate risk of bias
>12	low risk of bias

MINORS-Score (comparative studies)

<12	high risk of bias
12-18	moderate risk of bias
>18	low risk of bias

Supplement 6: Risk of Bias Analysis for non-randomized, case-control studies (Newcastle-Ottawa Quality Assessment Scale)

Author (year)	Selection	Comparability	Exposure	Risk of bias?
**Puyana et al. (2019)**	**	*	***	moderate
**Zhao et al. (2023)**	***	*	***	low

Interpretation:

Score (max 9 stars)

0-3	high risk of bias
4-6	moderate risk of bias
7-9	low risk of bias

Supplement 7: Risk of Bias Analysis for randomized controlled trials (Cochrane Risk of Bias Tool 2.0)

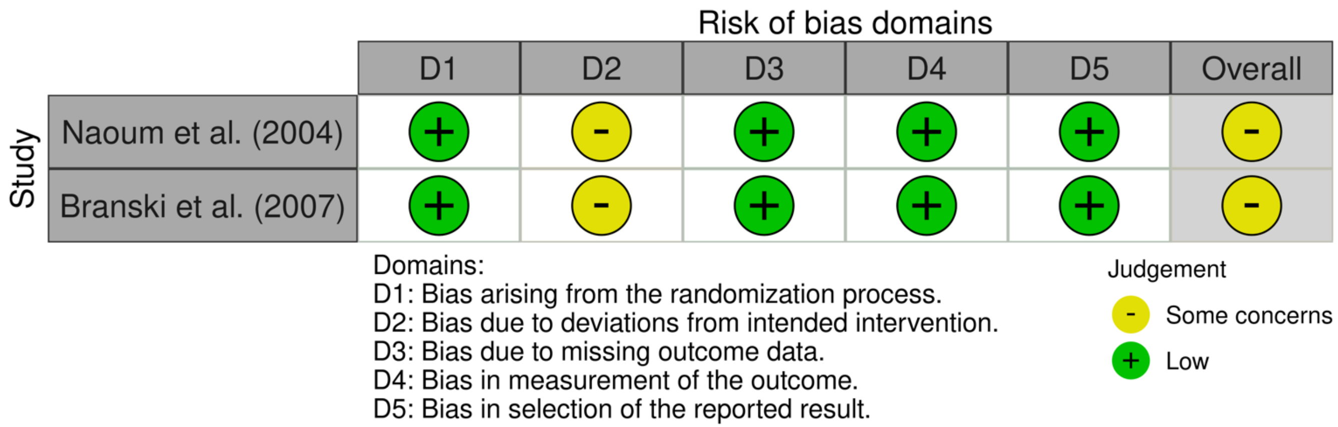

Supplement: Supplementary file 1 — Supplementary file1 (PDF 334 kb) [file 10561_2025_10161_MOESM1_ESM.pdf]
